# Lower urinary albumin‐to‐creatinine ratio predicted all‐cause and cardiovascular mortality in Chinese population with diabetes and prediabetes—The Shanghai Changfeng cohort study

**DOI:** 10.1111/1753-0407.13497

**Published:** 2023-11-20

**Authors:** Lingyan Chen, Li Wu, Qian Li, Hui Ma, Ting Liu, Jing Li, Baisheng Pan, Yu Hu, Huandong Lin, Xin Gao

**Affiliations:** ^1^ Department of Geriatrics, Zhongshan Hospital Fudan University Shanghai China; ^2^ Department of Endocrinology and Metabolism, Zhongshan Hospital Fudan University Shanghai China; ^3^ Clinical Laboratory, Zhongshan Hospital Fudan University Shanghai China; ^4^ Fudan Institute for Metabolic Diseases Shanghai China

**Keywords:** all‐cause mortality, cardiovascular mortality, diabetes mellitus, prediabetes mellitus, urinary albumin‐to‐creatinine ratio

## Abstract

**Introduction:**

Elevated urinary albumin‐to‐creatinine ratio (UACR) was associated with increased mortality in general population and diabetic patients. However, whether the association remains similar in the subjects with different status of glucose metabolism was unclear. The optimal level of UACR in predicting mortality also remained unknown. This study aims to investigate the relationship between UACR with all‐cause and cardiovascular mortality in population with different status of glucose metabolism and explore the predictive cutoff point of UACR.

**Methods:**

Six thousand three hundred and eighty‐six community‐dwelling individuals aged ≥45 years were enrolled and followed for an average of 5.3 years. Cox proportional hazards model was performed to analysis the association of baseline UACR and all‐cause as well as cardiovascular mortality according to the status of glucose metabolism. Receiver operating characteristic curve was plotted to explore the optimal predictive cutoff point of UACR.

**Results:**

With UACR increasing, both the prevalence of all‐cause and cardiovascular death increased. Cox analyses showed baseline UACR independently predicted the risk of all‐cause and cardiovascular mortality in the patients with prediabetes mellitus (pre‐DM) and diabetes mellitus (DM) but not in subjects with normal glucose tolerance (NGT). When divided by quartiles of UACR, the cumulative survival rate decreased acrossing the quartiles. Compared to the subjects with lowest quartile of UACR, participants with UACR ≥7.40 mg/gCr had a higher risk of all‐cause mortality, and participants with UACR ≥16.60 mg/gCr had an increased risk of cardiovascular mortality in all hyperglycemia subjects. The optimal predictive cutoff point of UACR was about 17 mg/gCr.

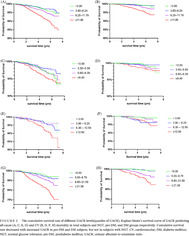

**Conclusion:**

UACR was an independent predictor of all‐cause and cardiovascular mortality in population with pre‐DM and DM but not in the subjects with NGT. The optimal predictive cutoff point of UACR is about 17 mg/gCr, which was far below the diagnostic cutoff point of microalbuminuria. Earlier interventions of albuminuria should be initiated from very early stage of hyperglycemia to reduce the burden of death in all patients whose glucose metabolism are impaired.

## Graphical abstract

**Highlights**

- The study firstly revealed that the relationship between UACR and mortality was different in subjects with different status of glucose metabolism. UACR was an important predictor of all‐cause and cardiovascular mortality in patients with pre‐DM and DM but not in subjects with normal glucose tolerance.
- The optimal cutoff point of UACR for prediction was about 17 mg/gCr, which was far below the current diagnostic cutoff point for microalbuminuria (30 mg/gCr).
- Earlier interventions of albuminuria should be initiated at much earlier stage of diabetic nephropathy, even at the normal stage of urine protein excretion, to ultimately reduce the burden of death in all the patients with hyperglycemia.

## 1 INTRODUCTION

Diabetes mellitus (DM) and its complications have contributed tremendously to the burden of mortality worldwide. The report of the International Diabetes Federation in 2021 estimated DM caused about 6.7 million deaths in adults aged 20–79 years (12.2%).^1^ Individuals with diabetes have a significantly higher risk of death than the general population. Observational studies reported two‐ to fourfold higher all‐cause and cardiovascular (CV) mortality in participants with DM as compared with those without DM.^2^ Among DM‐related death, CV mortality accounts for a large proportion.^3^, ^4^ Indeed, myocardial infarction is the leading cause of death among individuals with DM.^5^ Our previous study also found that the risk of all‐cause and CV mortality in diabetes was 1.91 and 2.03 times of that in people without diabetes, respectively.^6^ Even prediabetes (pre‐DM) was associated with an increased risk of all‐cause and CV mortality.^7^, ^8^ These suggest the importance of early prediction and intervention for mortality risk in individuals with impaired glucose regulation (IGR) including pre‐DM and DM.

According to the Kidney Disease Improving Global Outcomes chronic kidney disease (CKD) guideline, increased albuminuria was defined as urinary albumin‐to‐creatinine ratio (UACR) ≥30 mg/gCr, indicating kidney damage.^9^ Microalbuminuria was defined as UACR between 30 and 300 mg/gCr.^10^ It is generally believed that microalbuminuria is not only an indicator of glomerular endothelial damage representing microvascular injuries but also reflects widespread vascular damage.^11^ It is well established that microalbuminuria and macroalbuminuria were significant risk factors of CV and all‐cause mortality in diabetic patients.^12^, ^13^, ^14^, ^15^ The research of Hoorn Diabetes Care System cohort showed increased UACR (>3.0 mg/mmol) was prospectively associated with a higher risk of CV mortality in type 2 DM patients.^16^ The same phenomenon was also found in general population.^17^ However, a study by Siddique et al reported mildly increased albuminuria (UACR ≥10 and <30 mg/g) was a significant predictor of all‐cause and CV death in those with type 2 DM.^18^ This means that small elevations in UACR, even within normal range, were associated with increased mortality in diabetes. A few other studies also presented the similar results that low levels of albuminuria were associated with increased risk of all‐cause and CV mortality in general population.^19^, ^20^, ^21^ However, whether the association between UACR and the risk of mortality remains similar in different status of glucose metabolism was unknown. And what is the optimal threshold value of UACR predicting all‐cause and CV mortality? Whether and in which subpopulations we need to consider clinical interventions when individuals show mildly elevated UACR levels within the normal range?

In this study, we selected a relatively large cohort from Shanghai Changfeng Study^22^, ^23^ and aimed to investigate the value and optimal cutoff point of UACR in predicting all‐cause and CV mortality in a middle‐aged and elderly Chinese community population according to different status of glucose metabolism and to identify the high‐risk population susceptible to even mildly elevated UACR to prevent long‐term adverse health outcomes.

## 2 METHODS

### 2.1 Study population

From June 2009 to December 2012, 6511 individuals from Changfeng Community in Shanghai were enrolled in this cohort study. The details about the design of the study and the recruitment of subjects were described in our previous report.^22^ The inclusion criteria of participants were (1) aged 45 years or older, (2) lived in Shanghai Changfeng community for at least 5 years, and (3) volunteered to participate in the research. Sixty‐four individuals with incomplete body measurement data such as height, weight, and blood pressure, 19 subjects with missing biochemical data of blood and urine, 41 persons without examination of carotid ultrasound, and 1 person missing the vital status record during the follow‐up period were excluded. Finally, 6386 individuals were included in our final analyses (2712 male and 3674 female), including 511 (8.00%) subjects with cardiovascular disease (CVD) and 292 (4.57%) subjects with malignancy. This study was approved by the ethical committee of Zhongshan Hospital affiliated to Fudan University and all participants signed the informed consent.

### 2.2 Baseline measurements and covariates

Each participant underwent a standard questionnaire involving the information of medical history and lifestyle. They were instructed to maintain their regular lifestyle for at least 3 days before the examination. Height and weight were measured with the participants wearing only light clothes and without shoes. Meanwhile, waist and hip circumferences were both measured. Body mass index (BMI) was calculated as weight in kilograms divided by the square of height in meters (kg/m^2^). Resting blood pressure including systolic blood pressure (SBP) and diastolic blood pressure (DBP) was measured three times on the same arm with an electronic blood pressure monitor (OMRON Model HEM‐752 FUZZY, Omron Co., Dalian, China) after the participants had been sitting quietly for 5 min, and the average was calculated.

Blood samples were collected after a fasting period of at least 10 h. Fasting plasma glucose (FPG), total cholesterol (TC), triglycerides (TG), high‐density lipoprotein cholesterol (HDL‐C), low‐density lipoprotein cholesterol (LDL‐C), alanine aminotransferase (ALT), aspartate aminotransferase (AST), serum creatinine (Scr), and uric acid were measured using an automated bioanalyzer (HITACHI 7600, Tokyo, Japan). Estimated glomerular filtration rate (eGFR) was calculated by the formula of CKD‐Epidemiology Collaboration (EPI). The 2 h plasma glucose (2 h PG) was measured after taking a normal meal in people with a history of diabetes, and a 75 g oral glucose tolerance test was conducted in people without clear history of diabetes. Spot midstream urine specimens were collected from each individual in the morning. The urinary albumin concentration was determined by turbidimetric immunoassay and the urinary creatinine concentration was measured with a modified Jaffe method (model 7600 chemical analyzer; Hitachi, Tokyo, Japan). According to the conventional cutoff value,^9^ participants were categorized into three groups according to the levels of UACR: normalbuminuria (<30.0 mg/gCr), microalbuminuria (30.0–299.9 mg/gCr), and macroalbuminuria (≥300.0 mg/gCr).^10^ All blood samples were tested in the central laboratory of the Shanghai Zhongshan Hospital.

The carotid arteries were evaluated using a GE Logiq P5 (GE Healthcare, Milwaukee, WI, USA) scanner with a 10 MHz probe by a radiologist. Both sides of the carotid artery intima‐media thickness (CIMT) were measured in the common carotid artery approximately 1 cm proximal to the bifurcation at the far wall during end diastole. Three values were measured on each side, and the maximum CIMT values were used for the analysis. The maximum value of the left or right maximum CIMT ≥1.0 mm was defined as the CIMT thickening. Protruding lesions with a maximum CIMT ≥1.5 mm or CIMT greater than that of the surrounding medial membrane at least 0.5 mm was defined as carotid plaque.

Cigarette smoker was defined as smoking at least one cigarette per day for one or more years. Regular alcohol consumption was defined as the average alcohol consumption of more than 10 g of absolute alcohol per day for 1 year or longer. DM was defined as a FPG ≥7.0 mmol/L or 2‐hPG ≥11.1 mmol/L according to the World Health Organization 1999 criteria^24^ or a previous diagnosis of diabetes (self‐reported or using antidiabetic drugs. Pre‐DM was defined as FPG ≥6.1 and <7 mmol/L, and/or 2‐hPG ≥7.8 and <11.1 mmol/L.^24^ IGR included DM and pre‐DM. Hypertension was defined as a blood pressure ≥140/90 mm Hg or the self‐reported current use of antihypertensive medications.^25^ Dyslipidemia was defined as a TC ≥6.22 mmol/L, and/or TG ≥2.26 mmol/L, and/or LDL‐C ≥4.14 mmol/L, and/or HDL‐C <1.04 mmol/L, and/or a previous diagnosis or use of antilipidemic agents.^26^ Obesity was defined as BMI ≥28.0 kg/m^2^.^27^

### 2.3 Follow‐up and and mortality identification

We followed participants from the date of recruitment to the date of death or 31 December 2016, whichever came first. The mean follow‐up was 5.3 years. The vital status of participants was collected from the Shanghai Centers for Disease Control (SCDC), and the causes of death were coded according to the Tenth Revision of International Classification of Diseases (ICD‐10). The confirmation of causes from the SCDC was conducted by checking medical records. Causes of death during the follow‐up including malignant tumor (ICD‐10 codes C00‐C97), diseases of the heart (ICD‐10 codes I10‐I52), cerebrovascular diseases (ICD‐10 codes I60‐I69), DM (ICD‐10 codes E10‐E14), diseases of the respiratory system (ICD‐10 codes J00‐J99), diseases of the digestive system (ICD‐10 codes K00‐K93), dementia (ICD‐10 codes F00‐F03), and accidents (ICD‐10 codes V01‐X59). The main outcomes of this study were all‐cause and CV mortality. All‐cause mortality was defined as death due to any causes. CV mortality was defined as death caused by coronary disease, stroke, and peripheral arterial disease.

### 2.4 Statistical analysis

All statistical analyses were performed using SPSS software (version 19, IBM Corp., Armonk, NY) and Graphpad Prism software 9. Continuous variables were presented as means ± SD and categorical variables were presented as numbers and percentages. Baseline characteristics were compared between participants grouped by status of glucose metabolism or the degree of albuminuria. The one‐way analysis of variance was performed for continuous variables and the *χ* ^2^‐test was used for categorical variables. Cox proportional hazards model was performed to estimate the hazard ratio (HR) and 95% confidence interval (95% CI) of log‐transformed baseline UACR and other predictive factors for all‐cause and CV mortality, adjusting for age, gender, waist circumference (WC), SBP, ALT, FPG, TG, HDL‐C, eGFR, CIMT, cigarette smoking, and alcohol drinking. We introduced dummy variables in Cox analyses to further investigated association of baseline UACR and all‐cause and CV mortality. To clarify whether the relationship between baseline UACR and all‐cause and CV mortality varied according to the status of glucose regulation, we repeated the Cox analyses in subgroups categorized as NGT, pre‐DM, and DM. Furthermore, participants were divided into different groups according to the degree of albuminuria (<30, ≥30–300, and ≥300 mg/gCr) and the quartile of UACR. The HR and 95% CI of all‐cause and CV mortality in each group as compared with the reference group was calculated using multivariable‐adjusted Cox proportional hazards model. The Kaplan–Meier survival curve was plotted to compare the cumulative all‐cause and CV survival rates of different quartiles of UACR. Differences in incidences of CV and all‐cause mortality between groups were tested by log‐rank tests. Receiver operating characteristics (ROC) curve was plotted to investigate the optimal cutoff point of UACR in predicting all‐cause and CV mortality. *p* < .05 was considered statistically significant.

## 3 RESULTS

### 3.1 Baseline characteristics of the study population

Overall, the mean age of participants was 63.64 years (46.00 to 95.84 years), with 3557 NGT (55.7%), 1419 Pre‐DM (22.2%) and 1410 DM (22.1%). The prevalence of albuminuria at baseline was 10.1% (642), with 8.8% (561) microalbuminuria and 1.3% (81) macroalbuminuria respectively. The baseline characteristics of patients categorized by the status of glucose metabolism are shown in Table 1. With the status of glucose metabolism worsening, the subjects got older, and the levels of BMI, WC, hip circumference, SBP, DBP, FPG, TG, ALT, AST, Scr, and CIMT increased as well as the prevalence of obesity, hypertension, dyslipidemia, and carotid plaque, whereas the level of HDL‐C decreased. Meanwhile, the level of UACR and the prevalence of microalbuminuria and macroalbuminuria also ascended significantly (all *p* < .05). When the subjects were divided by the survival status, the results showed the patients in the dead group were much older and the levels of WC, SBP, FPG, Scr, UACR, and CIMT were higher when compared to the control group, whereas the level of eGFR was significantly lower. Meanwhile, the prevalence of obesity, hypertension, dyslipidemia, and carotid plaque, microalbuminuria and macroalbuminuria all increased (Table 2).

**TABLE 1:** Baseline characteristics and survival status at follow‐up of subjects according to diabetic status.

	Total (*n* = 6386)	NGT (*n* = 3557)	Pre‐DM (*n* = 1419)	DM (*n* = 1410)	*p* value
Age (years)	63.64 ± 9.71	61.85 ± 9.37	65.02 ± 9.72	66.76 ± 9.52	<.001
Men, *n* (%)	2712 (42.5)	1422 (40.0)	604 (42.6)	686 (48.7)	<.001
BMI (kg/m^2^)	24.26 ± 3.31	23.54 ± 3.12	24.85 ± 3.33	25.48 ± 3.27	<.001
WC (cm)	84.03 ± 9.67	81.63 ± 9.10	85.81 ± 9.70	88.30 ± 9.20	<.001
HC (cm)	93.32 ± 6.86	92.37 ± 6.49	94.10 ± 7.20	94.98 ± 7.02	<.001
SBP (mm Hg)	135.70 ± 19.33	131.08 ± 18.31	139.14 ± 19.03	143.88 ± 18.72	<.001
DBP (mm Hg)	76.28 ± 10.18	75.10 ± 9.91	77.66 ± 10.35	77.88 ± 10.31	<.001
ALT (u/L)	19.42 ± 7.19	17.59 ± 7.35	21.39 ± 9.34	22.07 ± 10.70	<.001
AST (u/L)	21.88 ± 8.77	21.18 ± 6.43	23.14 ± 10.89	22.38 ± 9.95	<.001
FPG (mmol/L)	5.62 ± 1.55	5.00 ± 0.42	5.42 ± 0.59	7.39 ± 2.43	<.001
TC (mmol/L)	5.07 ± 0.94	5.06 ± 0.92	5.13 ± 0.93	5.04 ± 0.99	.557
TG (mmol/L)	1.71 ± 1.24	1.51 ± 1.01	1.91 ± 1.51	2.00 ± 1.38	<.001
HDL‐C (mmol/L)	1.43 ± 0.38	1.49 ± 0.39	1.40 ± 0.39	1.32 ± 0.32	<.001
LDL‐C (mmol/L)	2.89 ± 0.80	2.90 ± 0.79	2.90 ± 0.78	2.85 ± 0.85	.098
Scr (μmol/L)	70.32 ± 20.72	70.05 ± 18.80	70.54 ± 22.89	70.79 ± 22.91	<.001
eGFR (mL/min/1.73m^2^)	93.68 ± 20.37	93.39 ± 19.11	93.11 ± 20.20	95.00 ± 23.3	.255
Lg‐UACR	0.87 ± 0.51	0.78 ± 0.45	0.87 ± 0.52	1.06 ± 0.59	.012
CIMT (mm)	0.89 ± 0.23	0.86 ± 0.20	0.90 ± 0.21	0.96 ± 0.30	<.001
Smokers, *n* (%)	1448 (22.7)	810 (22.8)	299 (21.1)	339 (24.0)	.562
Alcohol drinkers, *n* (%)	1058 (16.6)	569 (16.0)	241 (17.0)	248 (17.6)	.152
Existing diseases					
Obesity, *n* (%)	789 (12.4)	272 (7.6%)	215 (15.2)	302 (21.4)	<.001
Hypertension, *n* (%)	3542 (55.5)	1567 (44.1)	917 (64.6)	1058 (75.0)	<.001
Dyslipidemia, *n* (%)	2679 (42.0)	1219 (34.3)	687 (48.4)	773 (54.8)	<.001
Carotid plaque, *n* (%)	588 (9.2)	216 (6.1)	134 (9.4)	238 (16.9)	<.001
Albuminuria, *n* (%)	642 (10.1)	222 (6.2)	141 (9.9)	279 (19.8)	<.001
Microalbuminuria, *n* (%)	561 (8.8)	200 (5.6)	124 (8.7)	237 (16.8)	<.001
Macroalbuminuri, *n* (%)	81 (1.3)	22 (0.6)	17 (1.2)	42 (3.0)	<.001
Survival status at follow‐up					
All‐cause death, *n* (%)	356 (5.6)	127 (3.6)	76 (5.4)	153 (10.9)	<.001
CV death, *n* (%)	129 (2.0)	40 (1.1)	27 (1.9)	62 (4.4)	<.001

*Note*: Data are presented as mean ± SDs or as number (percentage).

Abbreviations: ALT, alanine aminotransferase; AST, aspartate aminotransferase; BMI, body mass index; CIMT, carotid intima‐media thickness; CV, cardiovascular; DBP, diastolic blood pressure; eGFR, estimate glomerular filtration rate; FPG, fasting plasma glucose; HC, hip circumference; HDL‐C, high‐density lipoprotein cholesterol; LDL‐C, low‐density lipoprotein cholesterol; Lg‐UACR, logarithm of UACR; SBP, systolic blood pressure; Scr, serum creatinine; TC, total cholesterol; TG, triglyceride; UACR, urinary albumin‐to‐creatinine ratio; WC, waist circumference.

**TABLE 2:** Baseline characteristics according to survival status at follow‐up.

	Alive (*n* = 6030)	Dead‐_all‐cause_ (*n* = 356)	*p* value	Non‐dead‐_CVD_ (*n* = 6257)	Dead‐_CVD_ (*n* = 129)	*p* value
Age (years)	63.04 ± 9.42	73.81 ± 8.78	<.001	63.39 ± 9.57	75.61 ± 8.85	<.001
Men, *n* (%)	2512 (41.6)	200 (56.2)	<.001	2644 (42.3)	68 (52.7)	.011
BMI (kg/m^2^)	24.25 ± 3.28	24.36 ± 3.74	.146	24.27 ± 3.30	24.68 ± 3.72	.149
WC (cm)	83.92 ± 9.63	85.84 ± 10.31	.001	83.97 ± 9.66	86.92 ± 10.08	.001
HC (cm)	93.32 ± 6.78	93.42 ± 8.12	.802	93.31 ± 6.84	94.43 ± 7.82	.065
SBP (mm Hg)	135.18 ± 19.02	144.33 ± 22.40	<.001	135.42 ± 19.13	149.10 ± 24.00	<.001
DBP (mm Hg)	76.35 ± 10.11	75.03 ± 11.32	.137	76.31 ± 10.13	74.96 ± 12.49	.137
ALT (u/L)	19.56 ± 8.36	17.16 ± 10.66	.095	19.50 ± 8.27	15.56 ± 8.88	.002
AST (u/L)	21.86 ± 8.71	22.16 ± 9.81	.529	21.90 ± 8.80	20.87 ± 7.62	.185
FPG (mmol/L)	5.58 ± 1.45	6.39 ± 2.63	<.001	5.61 ± 1.51	6.52 ± 2.79	<.001
TC (mmol/L)	5.08 ± 0.93	4.92 ± 0.97	.257	5.07 ± 0.94	4.95 ± 0.96	.156
TG (mmol/L)	1.72 ± 1.25	1.64 ± 1.14	.880	1.71 ± 1.29	1.82 ± 1.43	.317
HDL‐C (mmol/L)	1.43 ± 0.38	1.39 ± 0.36	.331	1.43 ± 0.38	1.36 ± 0.36	.039
LDL‐C (mmol/L)	2.89 ± 0.80	2.81 ± 0.84	.143	2.89 ± 0.80	2.80 ± 0.82	.212
Scr (μmol/L)	69.53 ± 18.79	83.75 ± 39.12	<.001	70.01 ± 20.08	85.44 ± 38.27	<.001
eGFR (mL/min/1.73m^2^)	94.30 ± 19.86	83.16 ± 25.40	<.001	93.96 ± 20.15	80.39 ± 25.72	<.001
Lg‐UACR	0.84 ± 0.49	1.23 ± 0.73	<.001	0.85 ± 0.50	1.43 ± 0.80	<.001
CIMT (mm)	0.88 ± 0.22	1.03 ± 0.32	<.001	0.88 ± 0.22	1.08 ± 0.34	<.001
Existing diseases						
Obesity, *n* (%)	728 (12.1)	61 (17.1)	.004	767 (12.3)	22 (17.1)	.071
Hypertension, *n* (%)	3275 (54.3)	267 (75.0)	<.001	3434 (54.9)	108 (83.7)	<.001
Diabetes, *n* (%)	1258 (20.9)	153 (43.0)	<.001	1349 (21.6)	62 (48.1)	<.001
Dyslipidemia, *n* (%)	2516 (41.7)	163 (45.8)	.136	2616 (41.8)	63 (48.8)	.066
Carotid plaque, *n* (%)	505 (8.4)	83 (23.3)	<.001	549 (8.8)	39 (30.2)	<.001
Albuminuria, *n* (%)	538 (8.9)	104 (29.2)	<.001	594 (9.5)	48 (37.2)	<.001
Microalbuminuria, *n* (%)	485 (8.0)	76 (21.3)	<.001	532 (8.5)	29 (22.5)	<.001
Macroalbuminuri, *n* (%)	53 (0.9)	28 (7.9)	<.001	62 (1.0)	19 (14.7)	<.001

*Note*: Data are presented as mean ± SDs or as number (percentage).

Abbreviations: ALT, alanine aminotransferase; AST, aspartate aminotransferase; BMI, body mass index; CIMT, carotid intima‐media thickness; CVD, cardiovascular disease; DBP, diastolic blood pressure; eGFR, estimate glomerular filtration rate; FPG, fasting plasma glucose; HC, hip circumference; HDL‐C, high‐density lipoprotein cholesterol; LDL‐C, low‐density lipoprotein cholesterol; Lg‐UACR, logarithm of UACR; NGT, normal glucose tolerance; SBP, systolic blood pressure; Scr, serum creatinine; TC, total cholesterol; TG, triglyceride; UACR, urinary albumin‐to‐creatinine ratio; WC, waist circumference.

### 3.2 Mortality during the follow‐up

**FIGURE 1:**
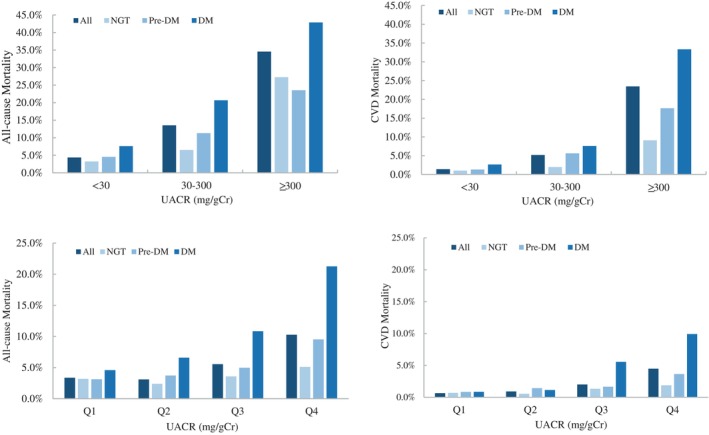
All‐cause and CV mortality at all follow‐up of subjects according to Baseline UACR. Both all‐cause and CV mortality increased with UACR elevating in total population and patients with different glucose metaolism. Quartiles of UACR: All: Q1 < 3.80 mg/gCr, Q2: 3.80–6.24 mg/gCr, Q3: 6.25–11.79 mg/gCr, Q4: ≥11.80 mg/gCr; NGT: Q1: <3.50 mg/gCr, Q2: 5.50–5.59 mg/gCr, Q3: 5.60–9.39 mg/gCr, Q4: ≥9.40 mg/gCr; pre‐DM: Q1: <3.90 mg/gCr, Q2: 3.90–6.29 mg/gCr, Q3: 6.30–12.59 mg/gCr, Q4: ≥12.60 mg/gCr; DM: Q1: <5.00 mg/gCr, Q2: 5.00–8.79 mg/gCr, Q3: 8.80–21.59 mg/gCr, Q4: ≥21.60 mg/gCr.). CV, cardiovascular; CVD, cardiovascular disease; DM, diabetes mellitus; NGT, normal glucose tolerance; pre‐DM, preiabetes mellitus; UACR, urinary albumin‐to‐creatinine ratio.

Mean follow‐up was 5.3 ± 2.1 years overall. A total of 356 deaths registered during the follow‐up, with a cumulative mortality of 5.6%. CV was the leading cause of death after malignancy (157/44.1%) accounted for 36.2% (129) of all deaths. With the status of glucose metabolism worsening, both all‐cause and CV mortality increased significantly. In the DM group, the incidence of all‐cause and CV death were up to 10.9% and 4.4%, respectively (Table 1). When divided by the level of UACR, we could find both all‐cause and CV mortality increased with UACR increasing no matter in NGT, pre‐DM, or DM groups. Patients with microalbuminuria and macroalbuminuria had significantly higher all‐cause and CV mortality than those with normoalbuminuria (Figure 1, Tables S1 and S2).

### 3.3 UACR and mortality risk

Cox proportional risk regression analysis showed that increased baseline UACR level was an independent risk factor for all‐cause and CV death in individuals with pre‐DM and DM, adjusting for gender, age, BMI, SBP, DBP, FPG, TG, LDL‐C, Scr, eGFR, CIMT, cigarette smoking, and alcohol drinking. Moreover, UACR was the strongest risk factor associated with all‐cause death (HR: 2.13, 95% CI: 1.65–2.76) in subjects with DM, and the second strongest risk factor for CV death in subjects with pre‐DM (HR: 2.40, 95% CI: 1.26–4.58) and DM (HR: 3.25, 95% CI: 2.14–4.74) (Table 3).

**TABLE 3:** Association of UACR with all‐cause mortality in Cox regression analysis.

	NGT			Pre‐DM			DM		
	HR	95% CI	*p* value	HR	95% CI	*p* value	HR	95% CI	*p* value
All‐cause mortality									
Gender (male)	1.48	0.93–2.36	.095	1.58	0.89–2.80	.116	0.86	0.57–1.29	.465
Age	1.11	1.08–1.13	<.001	1.07	1.04–1.11	<.001	1.12	1.10–1.15	<.001
BMI	1.03	0.97–1.09	.286	0.90	0.84–0.97	.006	0.97	0.92–1.01	.171
WC	1.46	0.99–2.13	.067	1.67	1.02–2.45	.042	1.33	1.00–2.98	.035
SBP	0.99	0.98–1.01	.294	0.99	0.98–1.01	.406	0.99	0.98–1.00	.193
DBP	1.01	0.98–1.03	.621	1.01	0.99–1.04	.376	1.01	0.99–1.03	.384
ALT	1.03	0.45–1.28	.315	1.19	0.78–1.93	.242	1.22	0.87–1.96	.179
FPG	1.14	0.75–1.73	.552	1.35	0.91–2.00	.138	1.09	1.03–1.16	.002
LDL‐C	0.95	0.76–1.19	.659	0.87	0.64–1.18	.365	0.96	0.79–1.17	.689
TG	0.75	0.56–1.01	.059	0.96	0.77–1.18	.672	0.92	0.80–1.06	.236
HDL‐C	0.89	0.67–1.23	.231	0.75	0.53–1.21	.097	0.73	0.51–1.02	.084
Scr	1.01	1.00–1.01	.060	1.00	0.99–1.01	.976	1.01	1.01–1.02	<.001
eGFR	0.99	0.98–1.01	.448	0.99	0.97–1.00	.149	1.01	1.00–1.03	.006
Lg‐UACR	1.28	0.88–1.85	.194	1.82	1.23–2.68	.003	2.13	1.65–2.76	<.001
CIMT	1.70	0.93–3.12	.084	2.63	1.28–5.41	.008	1.24	0.83–1.85	.287
Cigarette smoking	0.96	0.58–1.58	.863	1.43	0.72–2.86	.304	1.12	0.66–1.90	.661
Alcohol drinking	1.09	0.63–1.88	.761	0.66	0.31–1.39	.304	1.01	0.59–1.72	.970
CV mortality									
Gender (male)	1.72	0.75–3.95	.198	2.34	0.95–5.80	.065	0.56	0.28–1.12	.104
Age	1.14	1.09–1.20	<.001	1.05	0.99–1.11	.112	1.14	1.10–1.19	<.001
BMI	1.06	0.96–1.17	.276	0.89	0.78–1.00	.053	1.00	0.92–1.08	.942
WC	1.35	0.76–1.89	.186	1.62	1.02–2.69	.037	1.24	1.00–2.13	.006
SBP	1.00	0.97–1.02	.847	1.01	0.99–1.03	.367	1.00	0.99–1.02	.803
DBP	0.99	0.95–1.04	.668	0.99	0.95–1.04	.761	1.01	0.98–1.04	.379
ALT	0.97	0.36–1.51	.468	1.14	0.89–2.06	.069	1.57	1.01–2.38	.021
FPG	1.23	0.58–2.60	.583	1.00	0.50–1.98	.998	1.06	0.97–1.16	.223
LDL‐C	1.05	0.71–1.54	.810	0.50	0.29–0.87	.014	1.10	0.81–1.48	.554
TG	0.51	0.27–0.98	.044	1.08	0.88–1.33	.446	0.99	0.82–1.20	.939
HDL‐C	0.68	0.53–1.19	.182	0.73	0.61–1.00	.082	0.64	0.31–0.96	.031
Scr	1.00	0.99–1.02	.756	1.00	0.98–1.01	.722	1.01	1.00–1.02	.003
eGFR	0.99	0.96–1.01	.407	0.97	0.94–1.00	.072	1.02	1.00–1.03	.019
Lg‐UACR	1.45	0.77–2.71	.249	2.40	1.26–4.58	.008	3.25	2.14–4.74	<.001
CIMT	2.68	1.23–6.35	.025	3.81	1.43–10.18	.008	1.15	0.58–2.28	.680
Cigarette smoking	0.40	0.14–1.14	.088	0.72	0.20–2.60	.620	3.77	1.65–8.56	.002
Alcohol drinking	1.87	0.69–5.08	.221	0.32	0.06–1.80	.197	0.43	0.18–1.06	.060

Abbreviations: ALT, alanine transaminase; AST, aspartate transaminase; BMI, body mass index; CI, confidence interval; CIMT, carotid intima‐media thickness; CV, cardiovascular; DBP, diastolic blood pressure; DM, diabetes mellitus; eGFR, estimate glomerular filtration rate; FPG, fasting plasma glucose; HDL‐C, high‐density lipoprotein cholesterol; HR, hazard ratio; LDL‐C, low‐density lipoprotein cholesterol; NGT, normal glucose tolerance; pre‐DM, prediabetes; SBP, systolic blood pressure; Scr, serum creatinine; TG, triglyceride; UACR, urinary albumin‐to‐creatinine ratio.

**FIGURE 2:**
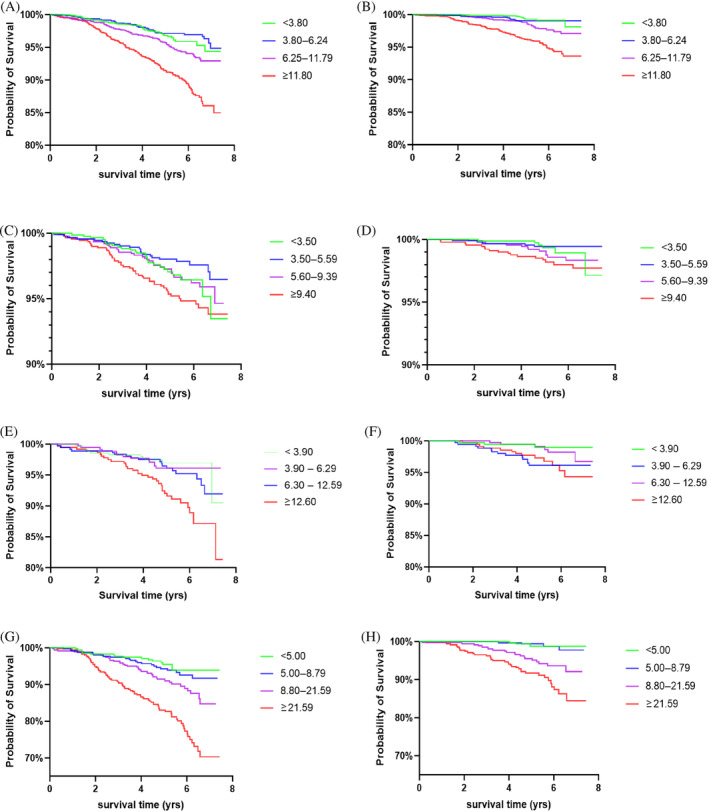
The cumulative survival rate of different UACR levels(quatiles of UACR). Kaplan–Meier's survival curve of UACR predicting all‐cause (A, C, E, G) and CV (B, D, F, H) mortality in total subjects and NGT, pre‐DM, and DM groups respectively. Cumulative survival rate decreased with increased UACR in pre‐DM and DM subjects, but not in subjects with NGT. CV, cardiovascular; DM, diabetes mellitus; Lg‐UACR, Logarithm of UACR; NGT, normal glucose tolerance; pre‐DM, prediabetes mellitus; UACR, urinary albumin‐to‐creatinine ratio.

To further analyze the association of baseline UACR and mortality, we introduced dummy variables in the analysis. Participants were divided into different groups according to the degree of albuminuria and the quartiles of UACR. Kaplan–Meier's survival analysis presented the cumulative survival rate decreased with increased UACR in Pre‐DM and DM subjects (Figure 2). As shown in Table 4, in total population, the risk of all‐cause and CV death in the microalbuminuria and macroalbuminuria groups was higher than that in normoalbuminuria group, and HR increased with albuminuria deteriorated. Further subgroup analysis showed there was no significant association between UACR and the risk of all‐cause and CV death in NGT subjects. But in pre‐DM subjects, the risk of all‐cause and CV death in macroalbuminuria group was 4.24‐ and 9.80‐fold that in normoalbuminuria group, respectively. In addition, when UACR was greater than 12.60 mg/gCr, the risk of all‐cause death increased. In DM subjects, microalbuminuria and macroalbuminuria were independent risk factors of all‐cause death, and macroalbuminuria was an independent risk factor of CV death. When UACR was greater than 8.80 mg/gCr, the risk of all‐cause and CV death already increased, with HR were 2.07 and 4.94 respectively. Further integrated analysis of subjects with IGR (pre‐DM + DM) showed the risk of all‐cause mortality already increased when UACR was greater than 7.40 mg/gCr, whereas the risk of all‐cause or CV death both significantly increased when UACR was greater than 16.60 mg/gCr, with HR were 2.57 and 3.61, respectively. The results suggested that the risk of all‐cause and CV death in patients with IGR increased with the elevation of UACR, even when the UACR was lower than 30 mg/gCr.

**TABLE 4:** Association of UACR and all‐cause and CV mortality in dummy variable analysis.

	All‐cause mortality			CV mortality		
	HR	95% CI	*p* value	HR	95% CI	*p* value
Total subjects						
UACR (mg/gCr)						
<30 (*n* = 5744)	1.00	—	—	1.00	—	—
30–300 (*n* = 561)	1.80	1.37–2.37	<.001	1.73	1.10–2.72	.018
≥300 (*n* = 81)	3.74	2.32–6.05	<.001	8.07	4.32–15.07	<.001
UACR quartile (mg/gCr)						
<3.80 (*n* = 1545)	1.00	—	—	1.00	—	—
3.80–6.24 (*n* = 1648)	0.96	0.65–1.41	.825	1.41	0.63–3.15	.400
6.25–11.79 (*n* = 1588)	1.44	1.02–2.05	.040	2.44	1.19–5.02	.015
≥11.80 (*n* = 1605)	1.91	1.37–2.66	<.001	3.42	1.71–6.82	<.001
Subjects of NGT						
UACR (mg/gCr)						
<30 (*n* = 3335)	1.00	—	—	1.00	—	—
30–300 (*n* = 200)	1.42	0.78–2.56	.251	1.23	0.42–3.64	.703
≥300 (*n* = 22)	2.95	0.81–10.77	.101	2.42	0.30–19.42	.405
UACR quartile (mg/gCr)						
<3.50 (*n* = 847)	1.00	—	—	1.00	—	—
3.50–5.59 (*n* = 917)	0.81	0.46–1.43	.463	0.93	0.28–3.10	.901
5.60–9.39 (*n* = 892)	1.10	0.65–1.86	.721	1.81	0.65–5.01	.254
≥9.40 (*n* = 901)	1.12	0.67–1.87	.663	1.70	0.63–4.60	.296
Subjects of pre‐DM						
UACR (mg/gCr)						
<30 (*n* = 1278)	1.00	—	—	1.00	—	—
30–300 (*n* = 124)	1.56	0.84–2.90	.160	1.99	0.75–5.27	.164
≥300 (*n* = 17)	4.24	1.21–14.83	.024	9.80	1.75–54.93	.009
UACR quartile (mg/gCr)						
<3.90 (*n* = 352)	1.00	—	—	1.00	—	—
3.90–6.29 (*n* = 348)	1.36	0.60–3.09	.458	1.95	0.45–8.45	.370
6.30–12.59 (*n* = 362)	1.91	0.88–4.14	.099	2.07	0.49–8.71	.320
≥12.60 (*n* = 357)	2.74	1.35–5.58	.005	2.70	0.73–10.01	.136
Subjects of DM						
UACR (mg/gCr)						
<30 (*n* = 1131)	1.00	—	—	1.00	—	—
30–300 (*n* = 237)	2.04	1.40–2.96	<.001	1.74	0.93–3.25	.081
≥300 (*n* = 42)	3.36	1.79–6.32	<.001	8.93	4.07–19.58	<.001
UACR quartile (mg/gCr)						
<5.00 (*n* = 348)	1.00	—	—	1.00	—	—
5.00–8.79 (*n* = 349)	1.26	0.67–2.40	.472	1.17	0.26–5.27	.834
8.80–21.59 (*n* = 360)	2.07	1.14–3.78	.017	4.94	1.45–16.84	.011
≥21.60 (*n* = 353)	3.10	1.74–5.52	<.001	5.93	1.76–20.00	.004
Subjects of IGR						
UACR (mg/gCr)						
<30 (*n* = 2409)	1.00	—	—	1.00	—	—
30–300 (*n* = 361)	1.94	1.41–2.66	<.001	1.96	1.16–3.30	.011
≥300 (*n* = 59)	4.01	2.35–6.81	<.001	9.46	4.76–18.78	<.001
UACR quartile (mg/gCr)						
<4.30 (*n* = 696)	1.00	—	—	1.00	—	—
4.30–7.39 (*n* = 709)	0.91	0.54–1.53	.722	0.67	0.23–1.94	.459
7.40–16.59 (*n* = 714)	1.69	1.07–2.68	.024	1.86	0.80–4.35	.150
≥16.60 (*n* = 710)	2.57	1.68–3.94	<.001	3.61	1.66–7.83	.001

*Note*: Adjusted for age, gender, WC, SBP, ALT, FPG, TG, HDL‐C, eGFR, CIMT, cigarette smoking, alcohol drinking.

Abbreviations: ALT, alanine transaminase; CI, confidence interval; CIMT, carotid intima‐media thickness; CV, cardiovascular; DM, diabetes mellitus; eGFR, estimate glomerular filtration rate; FPG, fasting plasma glucose; HDL‐C, high‐density lipoprotein cholesterol; HR, hazard ratio; IGR, impaired glucose regulation; NGT, normal glucose tolerence; pre‐DM, prediabetes; SBP, systolic blood pressure; TG, triglyceride; UACR, urinary albumin‐to‐creatinine ratio.

### 3.4 Optimal cutoff points of UACR in predicting all‐cause and CVD mortality

**FIGURE 3:**
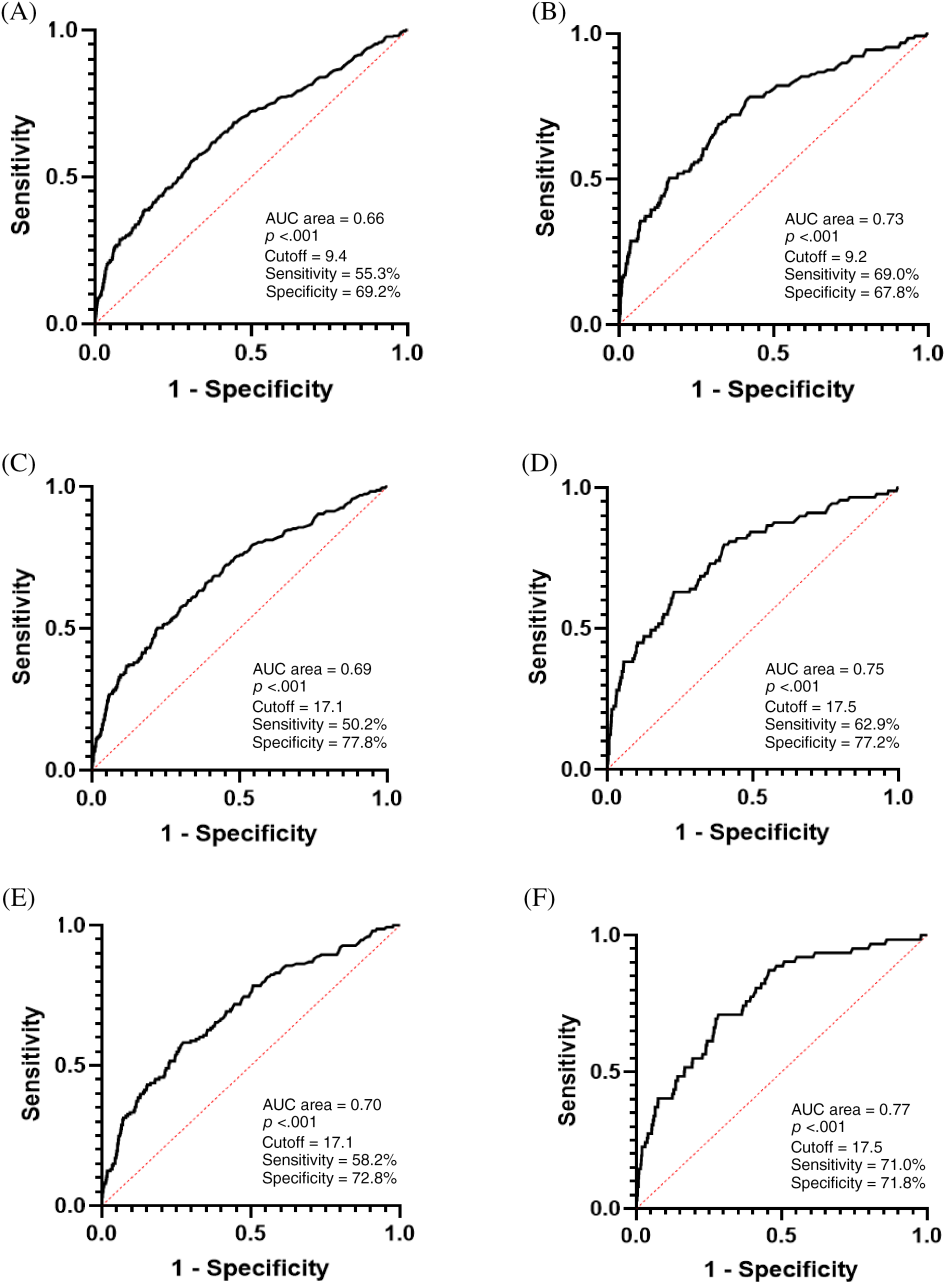
ROC curve of UACR predicting all‐cause and CV mortality in patients with different glucose metabolic status. ROC curve of UACR predicting all‐cause (A, C, E) and CV (B, D, F) mortality in total subjects, patients with IGR and patients with DM respectively. The results showed that both in patients with IGR and DM, the optimal cutoff points for UACR prediction of all‐cause and CV death was 17.1 and 17.5 mg/gCr, respectively. AUC, area under the curve; CV, cardiovascular; DM, diabetes mellitus; IGR, impaired glucose regulation; ROC, receiver operating characteristics; UACR, urinary albumin‐to‐creatinine ratio.

According to the results of Cox regression analysis, we further performed ROC analysis in pre‐DM and DM subjects. The results showed that the optimal cutoff points for UACR prediction of all‐cause and CV death was 17.1 and 17.5 mg/gCr, respectively, which were close to 17 mg/gCr and significantly lower than 30 mg/gCr, which is the cutoff point for diagnosis of microalbuminuria recommended by the current guidelines (Figure 3). The sensitivity of UACR at 17 mg/gCr in predicting all‐cause and CV death in IGR subjects was 50.2% and 62.9%, respectively, which were both higher than that of UACR at 30 mg/gCr. The Youden index of UACR at 17 mg/gCr in predicting all‐cause (0.278 vs. 0.243) and CV mortality (0.395 vs. 0.334) in individuals with IGR were higher than that of UACR at 30 mg/gCr (Table 5). Moreover, the sensitivity and Youden index both increased with glucose metabolism deterioration, no matter whether the cutoff point of UACR was at 17 or 30 mg/gCr, which indicated the predictive value of increasing urinary protein excretion was higher in DM patients, compared to NGT subjects.

**TABLE 5:** Sensitivity, specificity, and Youden index of different diagnostic cutoff points of UACR in predicting all‐cause and CV death.

	All‐cause mortality		CV mortality	
	Cutoff point (17 mg/gCr)	Cutoff point (30 mg/gCr)	Cutoff point (17 mg/gCr)	Cutoff point (30 mg/gCr)
Total subjects				
Sensitivity (%)	38.76	29.21	50.39	37.21
Specificity (%)	84.00	91.11	83.41	90.54
Youden index	0.228	0.203	0.338	0.277
Subjects of NGT				
Sensitivity (%)	18.11	14.96	22.50	15.00
Specificity (%)	88.83	94.11	88.71	93.89
Youden index	0.069	0.091	0.112	0.089
Subjects of pre‐DM				
Sensitivity (%)	34.21	23.68	44.44	37.04
Specificity (%)	82.43	90.84	82.04	90.59
Youden index	0.166	0.145	0.265	0.276
Subjects of DM				
Sensitivity (%)	58.17	43.79	70.97	51.61
Specificity (%)	72.47	83.21	70.99	81.75
Youden index	0.306	0.270	0.420	0.334
Subjects of IGR				
Sensitivity (%)	50.22	37.12	62.92	47.19
Specificity (%)	77.62	87.15	76.61	86.24
Youden index	0.278	0.243	0.395	0.334

Abbreviations: CV, cardiovascular; DM, diabetes mellitus; IGR, impaired glucose regulation; NGT, normal glucose tolerence; pre‐DM, prediabetes; UACR, urinary albumin‐to‐creatinine ratio.

## 4 DISCUSSION

In this prospectively observational cohort study, we found the level of UACR as well as the prevalence of albuminuria increased with the deterioration of glucose metabolism. Compared with the NGT population, IGR (pre‐DM and DM) individuals had significantly higher all‐cause and CV mortality, and UACR was an independent risk factor. More important, we found a lower level of UACR to predict all‐cause and CV death only in those IGR persons.

Similar to previous studies,^28^, ^29^ we found that UACR and SCr levels as well as the incidence of albuminuria and all‐cause and CV deaths all increased with glucose regulation disorder aggravating. Previous studies have confirmed renal failure, which appeared as decreased eGFR could independently predict all‐cause and CV mortality.^15^ In the present study, the level of eGFR was much lower in the dead group (no matter all‐cause or CV death), although the levels of Scr and eGFR were very similar among groups with different status of glucose metabolism. The prevalence of eGFR below 60 mL/min/1.73 m^2^ according to the formula of CKD‐EPI was only 4.7% (66), which was mainly concentrated in the DM population. Renal failure did not occur in the majority of the population. Therefore, eGFR and Scr were independent predictors for all‐cause and CV mortality only in the DM population. Notably, our research also identified UACR as an important independent predictor of all‐cause and CV mortality only in pre‐DM and DM but not in the population with NGT. Besides, both the risk of all‐cause and CV death caused by elevated UACR increased with the worsening of glucose regulation, with HR of all‐cause death increasing from 1.81 to 2.15 and HR of CV death increasing from 2.39 to 3.39 in pre‐DM and DM people respectively. Our results supported the previous studies,^15^, ^16^, ^17^, ^18^, ^30^ which indicated the close relationship between elevated UACR and all‐cause and CV mortality. However, most studies conformed the value of elevated UACR to predict all‐cause and CV mortality in diabetic and general population.^12^, ^13^, ^14^, ^15^, ^19^, ^20^, ^21^ To our knowledge, our study was the first study to compare the association between UACR and all‐cause or CV mortality in different status of glucose metabolism.

In order to explore the relationship between the severity of albuminuria with all‐cause and CV death, we further stratified UACR into normal, micro‐, and macro‐ albuminuria and found that the risk of all‐cause and CV death in patients with UACR≥30 mg/gCr was significantly increased (HR = 1.80 and 1.73 respectively), and the mortality of all‐cause and CV would be further increased in patients with UACR≥300 mg/gCr (HR = 3.74 and 8.07 respectively). Notably, this increased risk did not occur in people with NGT but in pre‐DM and DM patients. If combined pre‐DM and DM subjects (IGR), we found that UACR increased all‐cause and CV death when UACR≥30 mg/gCr (HR = 1.94 or 1.96 for all‐cause or CV death when UACR ≥30 mg/gCr and HR = 4.01 or 9.46 for all‐cause or CV death when UACR ≥300 mg/gCr). These results suggested that increased levels of UACR could predict all‐cause or CV mortality in all IGR patients. We should pay attention to urinary protein excretion even at the early stage of diabetes.

Previous studies, such as the Framingham Heart Study,^31^ reported low levels of urinary albumin excretion well below microalbuminuria threshold predicted the development of CV and all‐cause mortality in nonhypertensive, nondiabetic individuals. Is it possible that the all‐cause and CV mortality may have increased when the UACR level was lower than 30 mg/gCr, especially in people with abnormal glucose metabolism? The further UACR‐quartile analysis showed both the risk of all‐cause and CV death increased with UACR elevating. But the impact of UACR on all‐cause mortality seems bigger than that on CV mortality. This might be due to the reason that all‐cause mortality included multiple factors such as malignancy, not only cardiovascular disease. However, this is only a crude result that had not been adjusted, which needed further adjustment. The risk of all‐cause and CV death in total subjects has been significantly increased in the third quartile (UACR 6.25–11.79 mg/gCr) and further increased in the fourth quartile (UACR ≥11.80 mg/gCr). Similarly, this increased risk of death did not exist in the NGT group, but in the pre‐DM group, UACR ≥12.6 mg/gCr was associated with an increased risk of all‐cause death (HR = 2.74), whereas in the DM group, UACR >8.80 mg/gCr had increased the risk of all‐cause and CV death (HR = 2.07 and 4.94 respectively), and this risk further increased when it exceeded 21.60 mg/gCr (HR = 3.10 and 5.93 respectively). These results indicated that a significant increased risk of death happened when UACR was far below 30 mg/gCr. Our results first indicated that even mild elevation of UACR in normal range could increase the risk of all‐cause and CV death in all patients with hyperglycemia including pre‐DM and DM (UACR ≥7.40 mg/g to predict all‐cause mortality and UACR ≥16.60 mg/g to predict CV mortality). Our findings supported the notion that the contemporary threshold for microalbuminuria might be higher than the cutoff point at which increased mortality risk begins and challenged the designation of the range <30 mg/gCr as normoalbuminuria. A few previous studies reported albuminuria levels less than the traditional cutoff value (30 mg/gCr) predicted CV mortality in both general and diabetic population.^17^, ^20^, ^21^ The study by Zhang et al^21^ found that UACR levels of 10 to <30 mg/gCr were associated with the higher risk of all‐cause mortality in Chinese community population. The study of Siddique et al^18^ showed that mildly increased albuminuria (UACR ≥10 and <30 mg/gCr) was a significant predictor of all‐cause mortality in those with type 2 DM and stable coronary artery disease. Both the results reminded the risk of mortality already increased when UACR ≥10 mg/g. However, whether the results from these studies were similar in all the patients with hyperglycemia was unclear. Besides, these studies did not provide a clear cutoff point of UACR for predicting the risk of all‐cause and CV death, which makes it difficult and confused to the timing of initiating clinical intervention.

Therefore, in order to find out the optimal cutoff point of UACR to predict mortality, we performed ROC analysis. The results suggested that the optimal cutoff point for UACR to predict all‐cause and CV death was about 17 mg/gCr in both pre‐DM and DM subjects. Compared to the Youden index of conventional cutoff value of 30 mg/gCr, the Youden index of 17 mg/gCr for both all‐cause and CV mortality in pre‐DM and DM subjects were higher, which means UACR at 17 mg/gCr had better predictive value regarding to the risk of all‐cause and CV mortality. Although the levels of UACR in pre‐DM patients were lower than those in DM patients, the cutoff point of 17 mg/gCr still had predictive value. Compared to the Youden index of 17 mg/gCr for all‐cause mortality, the Youden index of 17 mg/gCr for CV mortality was higher. In addition, as we know, when AUC is 0.7–0.9, the ROC test has certain diagnostic accuracy. The area under the curve (AUC) of UACR at 17 mg/gCr predicting all‐cause mortality was 0.66–0.70, and the AUC of UACR at 17 mg/gCr predicting CV mortality was 0.73–0.77. According to AUC and Youden index, UACR at 17 mg/gCr might be more valuable in predicting CV mortality than all‐cause mortality. Nevertheless, all these factors indicated we should lower the cutoff point of UACR to 17 mg/gCr in all subjects with hyperglycemia to reduce mortality especially CV mortality. Furthermore, compared to cutoff point of 30 mg/gCr, although the specificity of UACR at 17 mg/gCr decreased slightly, the sensitivity increased and was highest in subjects with DM. This indicated that lowering UACR cut‐point was more beneficial for early screening of the population who had higher risk of death and the screening value increased with glucose disorder worsening. The results implied it might be more suitable that different cutoff points of UACR should be considered for different medical events, and UACR of 30 mg/gCr as a traditional cutoff point for diabetic nephropathy might be not appropriate for predicting all‐cause and CV death. Generally, all this strongly suggested that we should pay attention to the excretion of urinary protein even when UACR was far below 30 mg/gCr, especially in the patients with hyperglycemia. When UACR exceeds 17 mg/gCr, interventions should be needed to improve the prognosis of diabetes. Certainly, the establishment of the cutoff point of UACR needs to consider many factors, whereas we used the method of ROC curve only to formulate a rough cutoff from the perspective of mortality risk. Whether the cutoff point of 17 mg/gCr is suitable needs to be further verified in other prospective studies.

In the present study, using a large prospective cohort with long‐term follow‐up, we first compared the difference of association between UACR and the risk of mortality in different status of glucose metabolism and identified UACR as an important independent predictor of all‐cause and CV mortality in Chinese middle‐aged and elderly population with IGR but not in NGT subjects. The optimal cutoff point of UACR for prediction was ≈17 mg/gCr, which was far below the cutoff value of UACR to diagnose microalbuminuria. As an important indicator for the diagnosis of diabetic nephropathy, the results remind us that we should not only use UACR as a therapeutic target for kidney damage but also, more important, as an predictor of mortality. We should initiate the treatment of albuminuria‐reducing from the earlier stage of diabetic nephropathy in both pre‐DM and DM patients. Naturally, a few limitations need to be noticed in our study. First, we did not have autopsy data to confirm the causes of death, and this might induce some misclassification bias. However, in epidemiology studies this might be more practical. Second, albuminuria measurements show substantial day‐to‐day variability. In the present study, UACR value was available from only a single measurement, thus would result in imprecision in the UACR measurement. Third, a part of patients with proteinuria already started albuminuria reduction treatment, and the effect of medication on the outcome should be considered. More comprehensive history information should be collected in future studies. We analyzed only FPG in the current study. In the future, we will analyze more information about blood glucose such as HbA_1_C to determine its effect on the association between microalbuminuria and mortality. In addition, with aging and the duration of diabetes increasing, the number of people with decreased eGFR and increased excretion of urinary protein will be likely to increase in the future. We will collect more information to analyze their association with mortality. Finally, as the statistics of death were relatively based on the current follow‐up time in our study, the impact of some risk factors on mortality might not be yet apparent, such as dyslipidemia and hypertension.

## 5 CONCLUSION

Our study indicated that elevated UACR was an independent risk factor of all‐cause and CV mortality in Chinese middle‐aged and elderly population with IGR but not in NGT subjects. The optimal cutoff point of UACR for prediction was about 17 mg/gCr. We should pay attention to UACR even at the early stage of diabetes. Using UACR as a therapeutic target, the efficient interventions of albuminuria should be initiated from much earlier stage of diabetic nephropathy, which could improve the prognosis and ultimately reduce the burden of death in IGR.

## AUTHOR CONTRIBUTIONS

Lingyan Chen and Li Wu did the study design and literature research. Lingyan Chen wrote the manuscript and made critical editing. Lingyan Chen and Huandong Lin made definition of intellectual content and did data analysis. Lingyan Chen, Li Wu, Qian Li, Yu Hu, Hui Ma, Ting Liu, Jing Li, Baisheng Pan, Xin Gao, and Huandong Lin participated in the data acquisition and clinical study. Huandong Lin and Lingyan Chen contributed to statistical analysis. Huandong Lin and Yu Hu contributed to study concept and did the critical review and editing of the manuscript. Xin Gao and Huandong Lin were the guarantor of integrity of the entire study, as such, had full access to all the data in the study and take responsibility for the integrity of the data and the accuracy of the data analysis. All authors approved the final submitted version of the manuscript.

## FUNDING INFORMATION

This work was supported by the Shanghai Municipal Science and Technology Commission Foundation (Grant No. 16411954800); the Shanghai Municipal Science and Technology Major Project (Grant No. 2017SHZDZX01); and the key basic research grants from Science and Technology Commission of Shanghai Municipality (Grant No. 16JC1400500).

## DISCLOSURE

The authors have no conflicts of interest to declare.

## Supporting information


**Table S1.** All‐cause and CV death according to the level of UACR. Abbreviations: CV, cardiovascular; UACR, urinary albumin‐to‐creatinine ratio.
**Table S2.** All‐cause and CV death according to the quartiles of UACR. Abbreviations: CV, cardiovascular; UACR, urinary albumin‐to‐creatinine ratio.

## Data Availability

All data generated or analyzed during this study are included in this article and its supplementary material files. Further inquiries can be directed to the corresponding author.
