# Supplementary material for: Lower urinary albumin‐to‐creatinine ratio predicted all‐cause and cardiovascular mortality in Chinese population with diabetes and prediabetes—The Shanghai Changfeng cohort study
Source: J Diabetes. 2023 Nov 20;16(3):e13497. doi: 10.1111/1753-0407.13497 (PMC10925882; doi:10.1111/1753-0407.13497)
Supplement: Supplementary file 1 — Table S1. All‐cause and CV death according to the level of UACR. Abbreviations: CV, cardiovascular; UACR, urinary albumin‐to‐creatinine ratio. Table S2. All‐cause and CV death according to the quartiles of UACR. Abbreviations: CV, cardiovascular; UACR, urinary albumin‐to‐creatinine ratio. [file JDB-16-e13497-s001.docx]

**Supplementary Tables**

**Table S1 All-cause and CV death according to the level of UACR**

|  |  | **Total** | **<30mg/g** | **30-300mg/g** | **≥300mg/g** | **P value** |
| --- | --- | --- | --- | --- | --- | --- |
| **Total** | All-cause death, n (%) | 356 (5.6) | 252 (4.4) | 76 (13.5) | 28 (34.6) | <0.001 |
|  | CVD death, n (%) | 129 (2.0) | 81 (1.4) | 29 (5.2) | 19 (23.5) | <0.001 |
| **NGT** | All-cause death, n (%) | 127 (3.6) | 108 (3.2) | 13 (6.5) | 6 (27.3) | <0.001 |
|  | CVD death, n (%) | 40 (1.1) | 34 (1.0) | 4 (2.0) | 2 (9.1) | 0.001 |
| **Pre-DM** | All-cause death, n (%) | 76 (5.4) | 58 (4.5) | 14 (11.3) | 4 (23.5) | <0.001 |
|  | CVD death, n (%) | 27 (1.9) | 17 (1.3) | 7 (5.6) | 3 (17.6) | <0.001 |
| **DM** | All-cause death, n (%) | 153 (10.9) | 86 (7.6) | 49 (20.7) | 18 (42.9) | <0.001 |
|  | CVD death, n (%) | 62 (4.4) | 30 (2.7) | 18 (7.6) | 14 (33.3) | <0.001 |

**Table S2 All-cause and CV death according to the quartiles of UACR**

|  |  | **Q1** | **Q2** | **Q3** | **Q4** | **P value** |
| --- | --- | --- | --- | --- | --- | --- |
| **Total** | All-cause death, n (%) | 52 (3.4) | 51 (3.1) | 88 (5.5) | 165 (10.3) | <0.001 |
|  | CVD death, n (%) | 10 (0.6) | 15 (0.9) | 32 (2.0) | 72 (4.5) | <0.001 |
| **NGT** | All-cause death, n (%) | 27 (3.2) | 22 (2.4) | 32 (3.6) | 46 (5.1) | 0.017 |
|  | CVD death, n (%) | 6 (0.7) | 5 (0.5) | 12 (1.3) | 17 (1.9) | 0.027 |
| **Pre-DM** | All-cause death, n (%) | 11 (3.1) | 13 (3.7) | 18 (5.0) | 34 (9.5) | 0.001 |
|  | CVD death, n (%) | 3 (0.9) | 5 (1.4) | 6 (1.7) | 13(3.6) | 0.039 |
| **DM** | All-cause death, n (%) | 14 (4.0) | 16 (4.6) | 38 (10.6) | 85 (24.1) | <0.001 |
|  | CVD death, n (%) | 1 (0.02) | 5 (1.4) | 14 (3.9) | 42 (11.9) | <0.001 |

Total: Q1: <3.80mg/gCr; Q2: 3.80-6.24mg/gCr; Q3: 6.25-11.79mg/gCr; Q4: ≥11.80mg/gCr

NGT: Q1: <3.50mg/gCr; Q2: 5.50-5.59mg/gCr; Q3: 5.60-9.39mg/gCr; Q4: ≥9.40mg/gCr

Pre-DM: Q1: <3.90mg/gCr; Q2: 3.90-6.29mg/gCr; Q3: 6.30-12.59mg/gCr; Q4: ≥12.60mg/gCr

DM: Q1: <5.00mg/gCr; Q2: 5.00-8.79mg/gCr; Q3: 8.80-21.59mg/gCr; Q4: ≥21.60mg/gCr

UACR, urinary albumin-to-creatinine ratio; CV, cardiovascular; NGT, normal glucose tolerence; DM, diabetes mellitus
